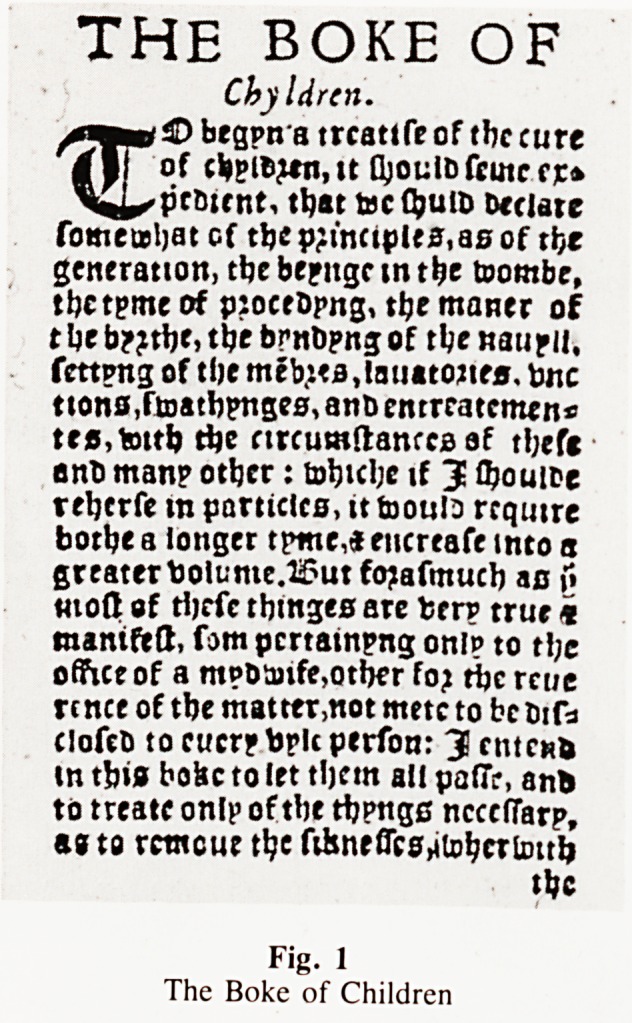# The Care of the Newborn in Antiquity

**Published:** 1991-12

**Authors:** Beryl Corner


### Care of the Newborn in Antiquity

**Beryl Corner, MD, FRCP**

In this talk 'Antiquity'' covered the earliest records till about 100 years ago when medical interest in perinatology began.

Birth has always been a subject for mysticism and religious practices. Since prehistoric times every community had a traditional birth attendant who was self-selected, illiterate and passed on her skill by word of mouth. These women still do most of the midwifery in the rural areas of the third world. The Old Testament contains the most authentic references which include the first in 1850 B.C., the instruction to Abraham that all Israeli male infants should be circumcised on the 8th day as a mark of the special relationship of the Hebrews and later Moses' law that mothers should present their sons in the temple at 40 days and daughters at 80 days for purification by the priest and animal sacrifice. In B.C. 1732 a midwife is mentioned as delivering Rachel after hard labour resulting in maternal death but the infant Benjamin was saved. Subsequently two midwives saved the whole race of Israel in Egypt by refusing to obey Pharaoh's command to kill all newborn infants on the grounds that the Hebrew women were strong and did not need the customary pressure on the abdomen to deliver. At Pharaoh's second attempt to kill the newborn boys the midwife saved the infant Moses.

In ancient times only mature, healthy infants were considered fit to survive; the premature and deformed were allowed to die from exposure to cold, starvation and even sacrifice by fire. Job refers to the practice of concealing premature babies till they died and this was usual in the civilisations of China, Sparta and Rome. Physicians were never involved in these decisions. Hippocrates only briefly mentions the newborn but suggests that they need special care and Aristotle describes milking blood back into the umbilical cord as a treatment for white asphyxia. Soranus and Galen of Ephesus emphasised skin care, breast feeding and swaddling to provide warmth and also to mould the limbs into good shape. The Arabic physicians in 600-700 A.D. rubbed the skin with nut oil and salt before swaddling.

THE BOKE OF
*Chyldren.*

TO begyn a treatiſe of the cure of chyldren, it ſhould ſeme expedient, that we ſhuld declare ſomewhat of the principles, as of the generation, the beynge in the wombe, the tyme of procedyng, the maner of the byrthe, the byndyng of the nauyll, ſettyng of the mēbres, lauatories, unctions, ſwathynges, and entreatementes, with the circumſtances of theſe and many other: whiche if I ſhoulde reherſe in particles, it would require bothe a longer tyme, & encreaſe into a greater volume. But forasmuch as ÿ moſt of theſe thinges are very true & manifeſt, ſom pertaininyng only to the office of a mydwife, other for the reuerence of the matter, not mete to be diſcloſed to euery vyle perſon: I entend in this boke to let them all paſſe, and to treate only of the thyngs neceſſary, as to remoue the ſiknesses, wherwith the

**Fig. 1**
The Boke of Children

Christianity spreading throughout Europe and the Middle East brought great changes in attitudes to maternal and infant care and the Nativity was a subject of great importance to the mediaeval artists. Gradually the Church became involved in saving the lives of the foetus and newborn infant. Strange practices were being followed by midwives which were vividly described by writers between A.D. 1500-1700. The witches' brew (Macbeth) contained ''finger of birth-strangled babe, ditch-delivered by a drab''. The first book in English on Obstetrics (Rösslein, 1540) describes the malpractices of the midwife as destroying the infant and depriving it of Holy Baptism and Phaire (1540) in the first English book of paediatrics states that the care of the newborn infant is a matter for the midwife and not the physician. Then the Church began to license midwives for a fee of 18 shillings. They were required to be of good moral character and instructed by the Parish priest in the administration of infant baptism.

The next developments took place mainly in France and Belgium when physicians became interested in Obstetrics; Mauriceau and Ambrose Paré set up training schools for midwifery in France and Holland. In England William Harvey and the Chamberlains tried to introduce care by physicians, dubbed man-midwives, and this was not accepted till the 18th century when the first maternity hospitals were opened in London and specialised in midwives' training. The first lectures were given by John Maubray in 1723 and the obstetricians Smellie and Denman followed. Their instruction included care of the newborn infant. Denham (1780) was the first obstetrician to attempt to save the life of a foetus by premature induction of labour by rupturing the membranes. Study of perinatal paediatrics made great advances in France in the nineteenth century and included ''the cold syndrome'', gavage feeding and intubation for asphyxia neonatorum and this culminated in the establishment in Paris of the first service for care of premature and feeble newborn infants by the obstetrician Budin in 1893 and the invention of the first incubator in 1881 designed by Tarnier, an obstetrician.

**REFERENCES**

BUDIN, P. (1907). The Nursling. Trans. W. Moloney. London.
DENMAN, T. (1795). Introduction to the Practise of Midwifery. London.
MAUBRAY, J. (1724). The Female Physician. Holland. London.
PHAIRE, T. (1540). The Boke of Children.
GENESIS, ch. 17, v.12.
ch. 35, v.17.
EXODUS, ch. 1, v. 16-121.
JOB, ch. 3, v.16.
RÖSSLEIN (1540) tr. The Byrth of Mankynde.

## Figures and Tables

**Fig. 1 f1:**